# Respiratory syncytial virus NS1 inhibits anti-viral Interferon-α-induced JAK/STAT signaling, by limiting the nuclear translocation of STAT1

**DOI:** 10.3389/fimmu.2024.1395809

**Published:** 2024-06-13

**Authors:** Claudia Efstathiou, Yamei Zhang, Shubhangi Kandwal, Darren Fayne, Eleanor J. Molloy, Nigel J. Stevenson

**Affiliations:** ^1^ Viral Immunology Group, Trinity Biomedical Sciences Institute, School of Biochemistry and Immunology, Trinity College Dublin, Dublin, Ireland; ^2^ Molecular Design Group, School of Chemical Sciences, Dublin City University, Glasnevin, Ireland; ^3^ Molecular Design Group, School of Biochemistry and Immunology, Trinity Biomedical Sciences Institute, Trinity College Dublin, Dublin, Ireland; ^4^ DCU Life Sciences Institute, Dublin City University, Dublin, Ireland; ^5^ Paediatrics, Trinity College, Dublin, Ireland; ^6^ Neonatology, Children’s Hospital Ireland at Tallaght, Dublin, Ireland; ^7^ Neonatology, Coombe Women’s and Infants University Hospital, Dublin, Ireland

**Keywords:** RSV, Interferon, JAK/STAT signaling, nuclear translocation, viral immune evasion

## Abstract

Human respiratory viruses are the most prevalent cause of disease in humans, with the highly infectious RSV being the leading cause of infant bronchiolitis and viral pneumonia. Responses to type I IFNs are the primary defense against viral infection. However, RSV proteins have been shown to antagonize type I IFN-mediated antiviral innate immunity, specifically dampening intracellular IFN signaling. Respiratory epithelial cells are the main target for RSV infection. In this study, we found RSV-NS1 interfered with the IFN-α JAK/STAT signaling pathway of epithelial cells. RSV-NS1 expression significantly enhanced IFN-α-mediated phosphorylation of STAT1, but not pSTAT2; and neither STAT1 nor STAT2 total protein levels were affected by RSV-NS1. However, expression of RSV-NS1 significantly reduced ISRE and GAS promoter activity and anti-viral IRG expression. Further mechanistic studies demonstrated RSV-NS1 bound STAT1, with protein modeling indicating a possible interaction site between STAT1 and RSV-NS1. Nuclear translocation of STAT1 was reduced in the presence of RSV-NS1. Additionally, STAT1’s interaction with the nuclear transport adapter protein, KPNA1, was also reduced, suggesting a mechanism by which RSV blocks STAT1 nuclear translocation. Indeed, reducing STAT1’s access to the nucleus may explain RSV’s suppression of IFN JAK/STAT promoter activation and antiviral gene induction. Taken together these results describe a novel mechanism by which RSV controls antiviral IFN-α JAK/STAT responses, which enhances our understanding of RSV’s respiratory disease progression.

## Introduction

The release of Interferons (IFNs) is part of the earliest responses to viral infection. IFNs act on the infected and surrounding cells to increase the expression of antiviral IFN response genes (IRGs) (1). The expression of IRGs makes cells less permissive to viral replication, thus limiting the spread of infection (2). To overcome the effect of IFNs, viruses have evolved a spectrum of mechanisms to limit these anti-viral responses.

IFN signaling is potentiated through the Janus Kinase/Signal Transducers and Activators of Transcription (JAK/STAT) pathway. Binding of IFN to its receptor results in a conformational change in its cytoplasmic tail, bringing JAKs into close contact with each other, allowing their auto- and trans-phosphorylation. These activated JAKs phosphorylate the receptor tails, creating docking sites for STATs. Once bound, STATs are phosphorylated by JAKs, allowing them to dissociate from the receptor and form dimers. STAT1:STAT2 heterodimers associate with IRF9, forming the Interferon stimulated gene factor 3 (ISGF3) transcription factor; while STAT1 homodimers form the GAGA-associated factor (GAF) transcription factor (3). These large transcription factors are then actively transported to the nucleus, where they bind gene promoter regions. The translocation of transcription factor molecules from the cytoplasm to nucleus is mediated by the nuclear pore complex (NPCs). Nuclear transport proteins bind the cargo molecules via a nuclear localization signal (NLS). The largest family of nuclear transport receptors are karyopherins (also known as importins), which are further divided into the karyopherin-α and the karyopherin-β subfamilies (4). Once transported to the nucleus, ISGF3 and GAF bind to IFN-stimulated response element (ISRE) and Gamma IFN activation site (GAS) promoter regions, respectively, causing the expression of anti-viral and inflammatory IRGs (5).

Many IRGs have a direct antiviral function, by targeting multiple points of the viral life cycle and enhancing viral detection; together these immune responses strengthen the antiviral response and limit viral replication. Indeed, polymorphisms in many IRGs have been linked with poor virus clearance, further highlighting the importance of the IFN response (6, 7).

IFNs are potent anti-viral effector molecules and their expression and activation are tightly regulated. IFN signaling initiates a classical negative-feedback loop which prevents excessive IRG induction, thereby preventing an uncontrolled and damaging cytokine storm. There are multiple negative regulatory proteins and processes of IFN signaling, including phosphatases, receptor gene down-regulation, receptor endocytosis, proteolytic degradation of the receptor and suppressor of cytokine signaling (SOCS) proteins. The IFN signaling pathway can also be regulated through post-translational modifications, such as dephosphorylation and ubiquitination (8, 9).

Viruses disrupt JAK/STAT signaling through a variety of mechanisms to evade host immune responses, thus enabling unhindered viral replication and increased disease severity. One strategy that viruses use to disrupt the JAK/STAT pathway is by directly targeting its components. For example, we have shown that Hepatitis C virus (HCV) and Human immunodeficiency virus (HIV) encode viral proteins that target STAT1 and STAT3 for degradation (10, 11). Another immunoregulatory strategy is the induction of negative regulators of the JAK/STAT pathway, such as SOCS and Ubiquitin Specific Peptidase 18 (USP18). SOCS family proteins can inhibit the activation of JAKs, receptor chains and promote proteasomal degradation of JAKs (12, 13). It has been shown that HCV core protein upregulated SOCS3 to block anti-viral IFN-α-induced STAT1 phosphorylation (14). USP18 competes with JAK1 for IFNAR2 receptor binding, reducing JAK1 and STAT activation (15, 16). HCV has been reported to upregulate USP18 to block the IFN-α response (17). Additionally, the IFN signaling pathway can also be regulated through phosphatases which remove activating phosphate groups from signaling molecules (such as the STATs) (18, 19). Epstein-Barr virus (EBV) has been shown to promote the recruitment of SHP1 phosphatase to STAT1 to inhibit its tyrosine phosphorylation (20).

RSV causes a significant disease burden in the global population, with an estimated 33.1 million cases each year, and is the leading cause of infant bronchiolitis and viral pneumonia (21). The RSV genome consists of 10 genes, producing 11 proteins, including fusion glycoprotein (F), attachment glycoprotein (G), small hydrophobic protein (SH), nucleoprotein (N), large RNA polymerase (L), phosphoprotein (P), matrix protein (M) and two non-structural (NS) proteins, NS1 and NS2 (22). The two NS proteins are key to suppression of the IFN response, with both NS1 and NS2 documented to downregulate STAT2 protein expression and reduced IFN-induced IRG induction in A549 and HEK293T cells (23, 24). This is also supported by a study showing that infection with RSV NS1/NS2 deletion mutants induced increased IFN-β mRNA levels, compared to wild-type RSV-infected cells (25). Additionally, RSV-NS1 has been shown to upregulate SOCS1 and SOCS3 and inhibit the IFN-inducible antiviral response in A549 cells (26).

While RSV-NS1 and -NS2 have been shown to target the JAK/STAT pathway in different ways, depending on the cell line type, however the mechanism by which RSV NS proteins inhibit the pathway in upper respiratory track epithelial cells has yet to be defined. Furthermore, while an effective RSV vaccine has recently been approved for use in older adults (27, 28) and monoclonal antibody treatments exist for infants (29–31), no curative treatment for RSV exists.

Since it has been established that RSV-NS1 protein acts as an IFN antagonist in HEK293T embryonic kidney cells, we sought to determine the mechanism by which RSV proteins target type I IFN signal transduction in the BEAS 2b bronchial epithelial cell line. Our study revealed that RSV-NS1 interacted with STAT1 and limited its translocation to the nucleus. Further examination revealed that RSV-NS1 hindered the interaction between STAT1 and the importin, KPNA1; which may explain reduced nuclear translocation of STAT1 and IFN-α-activated JAK/STAT signaling responses.

## Methods

### Cells culture

BEAS 2b cells were cultured under 37°C and 5% CO2 using Dulbecco’s minimal essential medium (DMEM) containing 10% foetal bovine serum (FBS), 1 μg/ml penicillin & streptomycin (P/S).

### Transfection

Cells were seeded into 6-well plates at a density of 2.5x10^6^ cells per well and grown in DMEM with 10% FBS and 1 μg/ml P/S. The following day cells were transfected with 1μg plasmid DNA (pCIneo, RSV-NS1 or RSV-NS2) using lipofectamine 2000 following the manufactures instructions. After 24h cells were treated as described and harvested for protein, RNA, or prepared for imaging.

### qRT-PCR

Total RNA was extracted from cells using TRIreagent (Sigma, USA) following manufacture instructions. RNA was converted to cDNA using the SensiFAST cDNA Synthesis kit (Bioline, UK). qRT-PCR was performed using SYBR-green (Bio-Rad, USA) following the kit instructions Data analysis was carried out using the 2−ΔΔct method. The relative expression of each result was calculated based on expression of the constitutively expressed housekeeping reference gene ribosomal protein 15 (*RPS15*). Primer sequences: *MxA* forward GGTGGTGGTCCCCAGTAATG, reverse ACCACGTCCACAACCTTGTCT, *USP18* forward TCGTGCCTGGCTCACATAAG, reverse CAACCAGGCCATGAGGGTAG, *PKR* forward TCTCAGCAGATACATCAGAGT, reverse TCGGAGTTGCCTCTTAAGACTGT, *ISG15* forward TCCTGCTGGTGGTGGACAA, reverse TTGTTATTCCTCACCAGGATGCT, *KPNA1* forward AGAGCGAGGCCTGAAATCAT, reverse GTTTCCCACAGCTCGCAAAG, *RPS15* forward CGGACCAAAGCGATCTCTTC, reverse CGCACTGTACAGCTGCATCA.

### Luciferase reporter assay

BEAS 2b cells cultured were transfected with either ISRE-luc or GAS-luc firefly luciferase reporter and pRL-TK Renilla luciferase reporter, together with plasmids expressing indicated proteins. After 24h cells were treated with IFN-α (SRP4594–100UG, Sigma-Aldrich) for 18h and lysed using 1X Passive lysis buffer (Promega, USA). Firefly and Renilla luciferase signals were quantified using Dual Luciferase Reporter Assay System. The firefly luciferase activity levels were normalized to the Renilla luciferase activity levels.

### Western blotting

Total protein was extracted from cells using RIPA buffer supplemented with phosphatase and protease inhibitors (Phenylmethylsulfonyl fluoride (PMSF), Na3VO4, Leupeptin, Dithiothreitol (DTT)) immediately prior to use. Protein lysates were run through 10–15% acrylamide gels and then transferred onto Polyvinylidene diflouride (PVDF) membrane. The PVDF membrane was incubated with primary antibody (pSTAT1, 9167, Cell Signalling Technology; pSTAT2 88410, Cell Signalling Technology; STAT1, 9172S, Cell Signalling Technology; STAT2, SC-476, Santa Cruz Biotechnologies; RSV-NS1, a kind gift from Prof. Mike Teng USF, USA (32); KPNA1, 18137–1-AP, Proteintech; β-actin, A5441-.2ML, Sigma-Aldrich) overnight at 4C. The membranes were incubated in the appropriate secondary antibody (anti-Rabbit, 11859140, Fisher Scientific or anti-Mouse, 10158113, Fisher Scientific) for 1h before imaging (Bio-Rad Imager). densitometry for each band was carried out using Bio-Rad Image Lab software (Bio-Rad, USA). Membranes were initially probed pSTAT1 or pSTAT2, and then reprobed with STAT2 or STAT1, respectively. As a result the pSTAT1 and STAT2 results and pSTAT2 and STAT1 results share the same β-actin bands.

### Immunoprecipitation

Cells were lysed in HEPES lysis buffer (50 mM HEPES, 150 mM NaCl, 2 mM EDTA, 1% NP40 and 0.5% sodium deoxycholate) supplemented with 1 mM PMSF, 1 mM Na3VO4, 5μg/ml leupeptin and 1 mM DTT. Lysates were immunoprecipitated with STAT1 (9172S, Cell Signalling Technology) and protein A/G agarose beads (Santa Cruz Biotechnologies) before immunoblotting for STAT1 (9172S, Cell Signalling Technology), RSV-NS1 (a gift from Prof Michael Teng, USF), KPNA1 (18137–1-AP, Proteintech), and β-actin (A5441, Sigma-Aldrich).

### Confocal microscopy

Cells were seeded onto glass slides and transfected for 24h as described above. The cells were then treated with IFN-α and fixed with 4% paraformaldehyde (PFA) for 20min. Cells were washed with PBS and permeabilized with 0.2% Triton X-100 for 30min and blocked in 0.5% BSA for 1 hour at room temperature. The slides were treated with diluted primary antibodies (RSV-NS1, a gift from Prof Michael Teng, USF); STAT1, AHO0832, Thermo Fisher and incubated overnight at 4°C. These were then washed and incubated in secondary antibodies (anti-Rabbit, SAB4600084, Merck or anti-Mouse, 405322, MSC) for 1 hour in the dark at RT. The slides were mounted using DAPI ProGold Mounting media (P36941, Thermo Fisher) and imaged using a Lecia SP8 scanning confocal microscope. Quantitative analysis was performed using IMARIS software (Oxford Instruments).

### 3D protein-protein modeling

For predicting the molecular interactions between RSV-NS1 and STAT1, protein-protein docking was performed using Molecular Operating Environment (MOE) 2022.02 (33). The X-ray crystal structure selected for STAT1 (PDB: 1YVL) had a resolution of 3 Å (34). This structure was selected as it had the highest number of resolved amino acids i.e., 683 AA compared to the other STAT1 X-ray structures. On the other hand, there was only one X-ray crystal structure available for RSV-NS1 (PDB: 5VJ2) which had a resolution of 2.22 Å (35) and was 135 AA in length.

The protein structures were prepared using MOE quick prep option with the default Amber10:EHT force field in order to consider explicit hydrogen atoms, tautomeric states and possible breaks in protein structure prior to conducting restrained all atom molecular mechanics minimization and electrostatics calculations. The prepared protein structures were then used for performing MOE protein-protein docking with patch analysis set to use a hydrophobic patch potential. The number of poses were set to 10,000 and 100 for pre-placement and refinement respectively. The docking was performed in triplicate to check reproducibility of the docking algorithm used in MOE. To compare the most stable docked pose predicted by MOE, we also performed docking with the same prepared protein files on the ClusPro 2.0 server (36) and the ZDOCK 3.0.2 server (37) with default values.

Lastly, MOE contact analysis was used to analyze the interactions between the best scored docked RSV-NS1 and STAT1 poses from MOE and the two servers. In MOE contact analysis six types of contacts can be identified: Hydrogen bonds (Hbond), Metal, Ionic, Arene, Covalent, and Van der Waals distance interactions (Distance). All of these options were selected, and the calculations were performed by setting the display and within option to ALL. The output of each contact analysis was then compared to identify the common amino acid hotspots from the three best docked poses predicted by three different docking methods.

### Statistical analysis

Statistical comparisons between groups were performed using GraphPad Prism statistical analysis software (version 9). Data is represented as the mean ± SD unless otherwise stated.

## Results

### RSV-NS1 antagonizes type I IFN signaling in the BEAS 2b epithelial cell line

**Figure 1:**
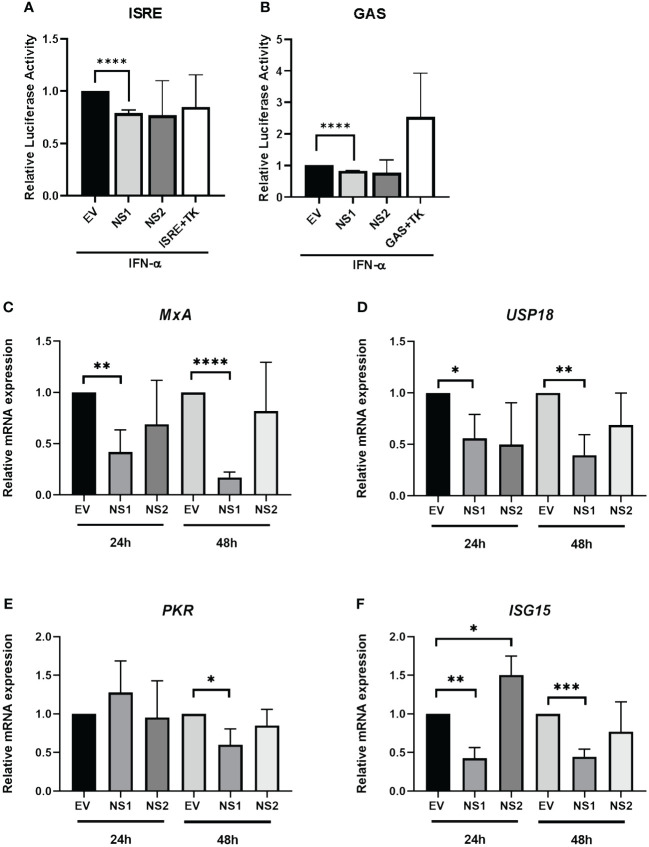
RSV-NS1 suppresses Type I IFN promoter activity and IRG expression in the BEAS 2b epithelial cell line. BEAS 2b cells were transfected EV, RSV-NS1 or RSV-NS2, along with ISRE-Luc or GAS-Luc and TK Renilla. At 24h post-transfection, cells were stimulated by 1000U/mL of IFN-α. Dual luciferase activity was measured 18h post-IFN-α treatment for levels of **(A)** ISRE and **(B)** GAS (n=4). BEAS 2b cells were transfected with EV, NS1 or NS2 for 24h or 48h. Cells were extracted for total RNA, before analyzing mRNA levels of **(C)** *MxA*, **(D)** *UPS18*, **(E)** *PKR* and **(F)** *ISG15* (n=3). Gene expression was calculated relative to *RPS15* and compared to EV controls, which were normalized to 1. All data is shown as mean ± SD. Significance was determined by unpaired t-test *p<0.05, **p<0.01, ***p<0.001, ****p<0.0001.

Having previously observed that RSV-NS1/NS2 promoted STAT2 degradation in HEK293T cells (24), we initially wanted to examine the effect of RSV NS proteins on anti-viral and inflammatory gene promoter activity in an upper respiratory epithelial cell line. Therefore, we expressed RSV-NS1 or RSV-NS2 in epithelial BEAS 2b cells and measured their effect upon IFN-α-induced ISRE and GAS activity using a luciferase reporter assay. BEAS 2b cells were transfected with EV control or plasmids expressing RSV-NS1 or RSV-NS2 with ISRE-luc or GAS-luc, along with a control pRL-TK plasmid. After 24h, the cells were stimulated with IFN-α for 18h. We observed that the expression of RSV-NS1, but not RSV-NS2, significantly reduced both IFN-α-induced ISRE and GAS promoter activation, suggesting that RSV’s NS1 protein suppresses the IFN-α JAK/STAT signaling pathway in bronchial epithelial cells (**Figures 1A, B**).

### RSV-NS1 reduces IRG expression in the BEAS 2b epithelial cell line

Having observed a reduction of IFN-α-mediated ISRE and GAS activity in BEAS 2b cells expressing RSV-NS1, we subsequently investigated if RSV-NS1 affected anti-viral IRG expression. BEAS 2b cells were transfected with EV, RSV-NS1 or RSV-NS2 for 24h or 48h, before measuring mRNA expression of the IRGs, *MxA, USP18, PKR* and *ISG15*. While RSV-NS1 expression significantly reduced *MxA, USP18* and *ISG15* mRNA expression at both 24h and 48h (**Figures 1C, D, F**), *PKR* was significantly reduced at only 48h. Furthermore, RSV-NS2 expression had no effect upon *MxA, USP18* nor *PKR* at 24h or 48h; while *ISG15* was increased by RSV-NS2 at 24h, but there was no effect at 48h (**Figure 1E**). Together with our ISRE and GAS luciferase reporter results (**Figures 1A, B**), these findings further confirm that RSV-NS1, but not RSV-NS2, inhibits ISRE and GAS promoter activity and IRG expression in the bronchial epithelial BEAS 2b cell line.

### RSV-NS1 enhances phosphorylation of STAT1 in the BEAS 2b epithelial cell line

**Figure 2:**
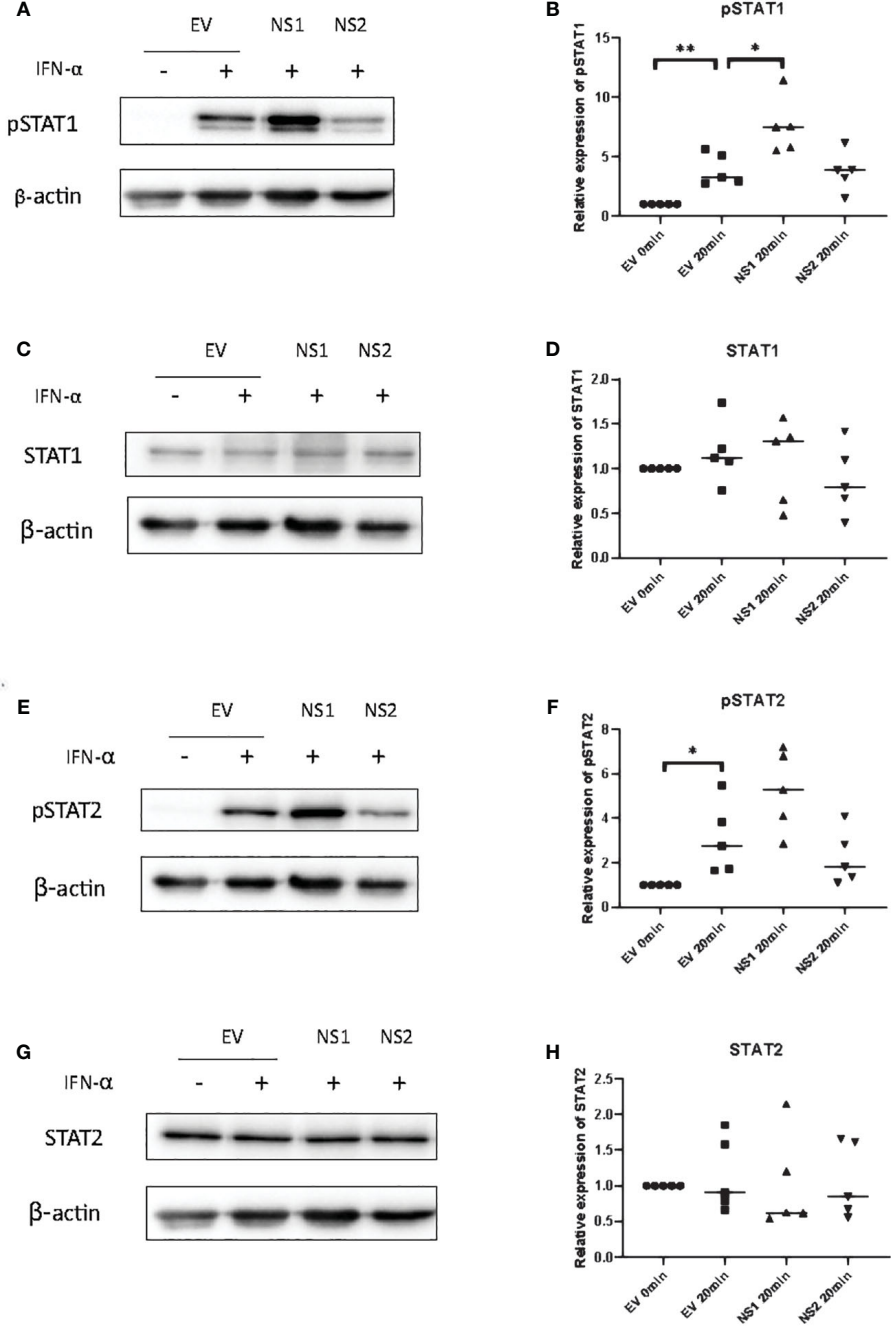
RSV-NS1 enhances IFN-α-induced STAT1 phosphorylation in the BEAS 2b epithelial cell line. BEAS 2b cells were transfected with EV, RSV-NS1 or RSV-NS2 for 24h and treated with 1000IU IFN-α for 20min. Cell lysates were collected and levels of **(A)** pSTAT1, **(C)** STAT1, **(E)** pSTAT2 and **(G)** STAT2 were measured by western blotting (n=5). Densitometry of **(B)** pSTAT1, **(D)** STAT1, **(F)** pSTAT2 and **(H)** STAT2 was performed using Image Lab software and values for STATs or phosphorylated STATs were calculated relative to β-actin and compared to the EV transfected untreated control, which was normalized to 1. (N.B pSTAT1 and STAT2 were probed in one membrane and therefore share the same β-actin, and the pSTAT2 and STAT1 were probed in one membrane and therefore share the same β-actin). Data is presented as mean ± SD. Statistics calculated by unpaired t test *p<0.05 and **p < 0.01.

Since ISRE or GAS promoter activity are controlled by ISGF3 and GAF, respectively (38, 39), we hypothesized that the RSV-NS1-mediated reduction in total or phosphorylated STAT1/2 protein could be responsible for the observed reduction in downstream promoter activity and IRG expression in BEAS 2b cells. Therefore, to identify if the expression of RSV-NS1 limits total STAT expression and/or STAT phosphorylation, BEAS 2b cells were transfected with RSV-NS1, RSV-NS2 or EV for 24h. Following this, cells were treated with IFN-α for 20min and levels of pSTAT1, STAT1, pSTAT2 and STAT2 were measured by western blotting. To our surprise pSTAT1 was significantly increased upon expression of RSV-NS1 (**Figures 2A, B**). While western blot analysis visually indicated an increase in pSTAT2 levels upon expression of RSV-NS1, densitometric analysis, using the loading control, revealed this was not statistically significant (**Figures 2E, F**). In contrast, densitometric analysis revealed that RSV-NS2 had no effect upon either pSTAT1 nor pSTAT2 levels (**Figures 2A, B, E, F**). Neither NS1, nor NS2 expression significantly affected total STAT1 (**Figures 2C, D**) or STAT2 (**Figures 2G, H**) protein levels.

### RSV-NS1 limits nuclear translocation of STAT1 in the BEAS 2b epithelial cell line

**Figure 3:**
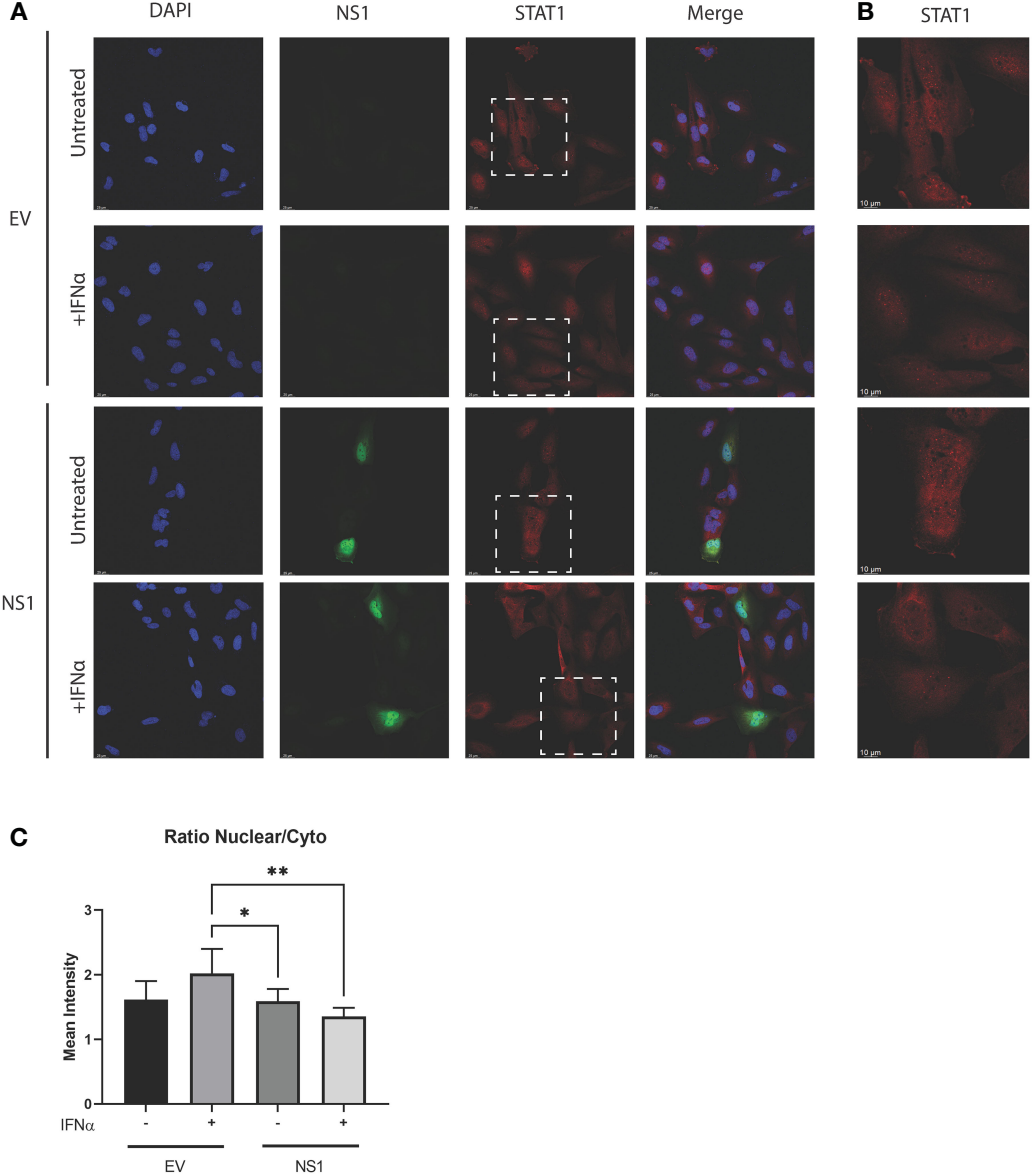
Expression of RSV-NS1 limits nuclear translocation of STAT1. BEAS 2b cells were transfected RSV-NS1 or EV and stimulated with or without (UT) 1000IU IFN-α for 30min. **(A)** Cells were stained for RSV-NS1, STAT1 and DAPI, and visualized using confocal microscopy. Images are representative of three independent experiments. Scale measurement bar represents 25μm in length. **(B)** STAT1 is highlighted within the dashed box and shown at higher magnification. Scale measurement bar represents 10μm in length. Quantification of STAT1 intensity in the nucleus and cytoplasm was determined using IMARIS software and **(C)** the ratio of nuclear to cytoplasmic STAT1 intensity was determined. All data is shown as mean ± SD. Significance was determined by One-way ANOVA multiple comparison test (n=3) *p < 0.05 and **p < 0.01.

The statistically significant enhancement of pSTAT1 by RSV-NS1 (**Figure 2**), would normally be associated with increased ISRE and GAS promoter activity and IRG expression. However, we showed that RSV-NS1 expression reduced both these downstream pathways read outs in the BEAS 2b epithelial cell line (**Figure 1**). Therefore, we next assessed whether RSV-NS1 was hindering the migration of STAT1 to the nucleus and thus limiting its transcriptional activity. BEAS 2b cells were transfected for 24h with RSV-NS1 or EV, before stimulating with 1000IU/ml IFN-α for 30min. To quantify the confocal microscopy observations the ratio of nuclear to cytoplasmic STAT1 were determined using IMARIS software. Immunofluorescence analysis (**Figures 3A, B**) confirmed that the ratio of nuclear to cytoplasmic STAT1 was significantly reduced upon expression of RSV-NS1, compared to IFN-α treated EV transfected cells (**Figures 3A–C**), suggesting that NS1 restricted STAT1 nuclear translocation. Altogether, these results indicate that RSV-NS1 inhibits IFN-α-mediated ISRE and GAS induction and IRG expression, by suppressing STAT1 nuclear translocation in the bronchial BEAS 2b epithelial cell line.

### RSV-NS1 has no effect upon KPNA1 protein expression

**Figure 4:**
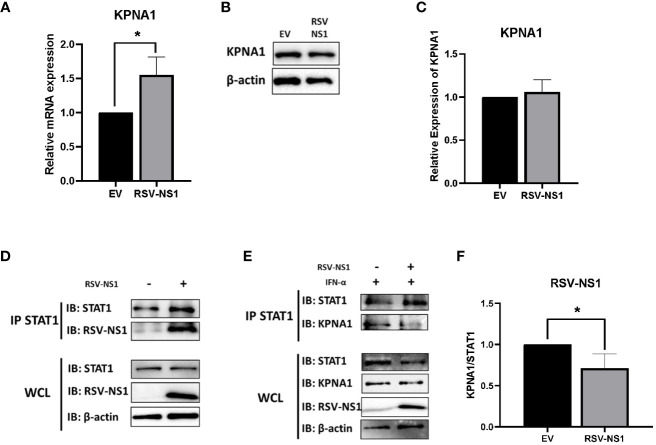
RSV-NS1 associates with STAT1, while KPNA1:STAT1 interaction is reduced. BEAS 2b cells were transfected with RSV-NS1 or EV for 24h. **(A)** Total RNA was extracted before analyzing mRNA levels of KPNA1, gene expression was calculated relative to the RPS15 and compared to EV, which was normalized to 1. BEAS 2b cells were transfected with RSV-NS1 or EV for 24h. **(B)** Protein was extracted before analyzing protein levels of KPNA1. Densitometry of **(C)** KPNA1 was performed using Image Lab software and values for KPNA1 were calculated relative to β-actin and compared to EV, which was normalized to 1 (n=4). **(D)** BEAS 2b cells were transfected with RSV-NS1 or EV for 24h. Lysates were immunoprecipitated (IPed) using a STAT1 antibody. IP and whole cell lysates (WCL) were subjected to Western blotting using STAT1, RSV-NS1 and β-actin antibodies. **(E)** BEAS 2b cells transfected with RSV-NS1 or EV for 24 h and stimulated with 1000IU IFN-α for 30min. Cells were lysed and immunoprecipitated using STAT1 antibody. IP and WCL were subject to Western blotting using STAT1, KPNA1, RSV-NS1 and β-actin. **(F)** Densitometric analysis was performed using Image Lab software and values of KPNA1 were calculated relative to STAT1 (IP) and compared to EV, which was normalized to 1 (n=4). Data is shown as mean ± SD. Significance was determined by unpaired t-test. *p < 0.05.

Karyopherin-α1 (KPNA1, also called importin-α5) is known to mediate the nuclear import of ISGF3 and is a critical importin involved in STAT1 nuclear translocation (40). Several viruses have been shown to limit the activity of karyopherins, thus stunting signaling pathway transduction. The porcine reproductive and respiratory syndrome virus (PRRSV) protein, Nsp1β, and the Foot Mouth disease virus (FMDV) 3C protease, have both been shown to degrade KPNA1 protein through the proteosome, which in turn, blocks STAT1 nuclear translocation (41, 42). Having observed that RSV-NS1 limits STAT1 nuclear translocation, we hypothesized that RSV may also be targeting expression of the nuclear transport protein, KPNA1. To explore this, mRNA and protein levels of KPNA1 were measured in BEAS 2b cells transfected with EV or RSV-NS1. We found that while expression of RSV-NS1 significantly increased *KPNA1* mRNA level (**Figure 4A**), it had no effect upon KPNA1 protein expression (**Figures 4B, C**), indicating that RSV-NS1 does not target KPNA1 for degradation.

### RSV-NS1 interacts with STAT1, while KPNA1 and STAT1 interaction is reduced

Having found that RSV-NS1 did not affect KPNA1 expression, we next measured the interaction between RSV-NS1, STAT1 and KPNA1, to analyze if RSV-NS1 might be reducing STAT1 nuclear translocation via restriction of the KPNA1:STAT1 interaction. BEAS 2b cells were transfected with EV or RSV-NS1 for 24h, before cells lysates were immunoprecipitated with anti-STAT1 antibody and immunoblotted for RSV-NS1. We found that RSV-NS1 interacted with STAT1 (**Figure 4D**), leading us to next hypothesize that RSV-NS1 may competitively bind to STAT1, reducing KPNA1:STAT1 interaction, which in turn suppresses STAT1 nuclear translocation. To test this theory, BEAS 2b cells were transfected with EV or RSV-NS1, followed by IFN-α treatment, before a co-IP assay, with anti-STAT1 antibody, was carried out. As shown in **Figures 4E, F**, in the absence of RSV-NS1, KPNA1 clearly coimmunoprecipitated with STAT1 after IFN-α stimulation. However, the KPNA1:STAT1 interaction was significantly reduced in the presence of RSV-NS1 (**Figures 4E, F**), suggesting that RSV-NS1 impaired the binding between STAT1 and KPNA1. These findings reveal a novel immune evasion mechanism by which RSV-NS1 blocks nuclear translocation of STAT1 and thus suppresses anti-viral responses to IFN-α.

### 3D protein-protein modeling shows interaction between RSV-NS1 and STAT1

**Figure 5:**
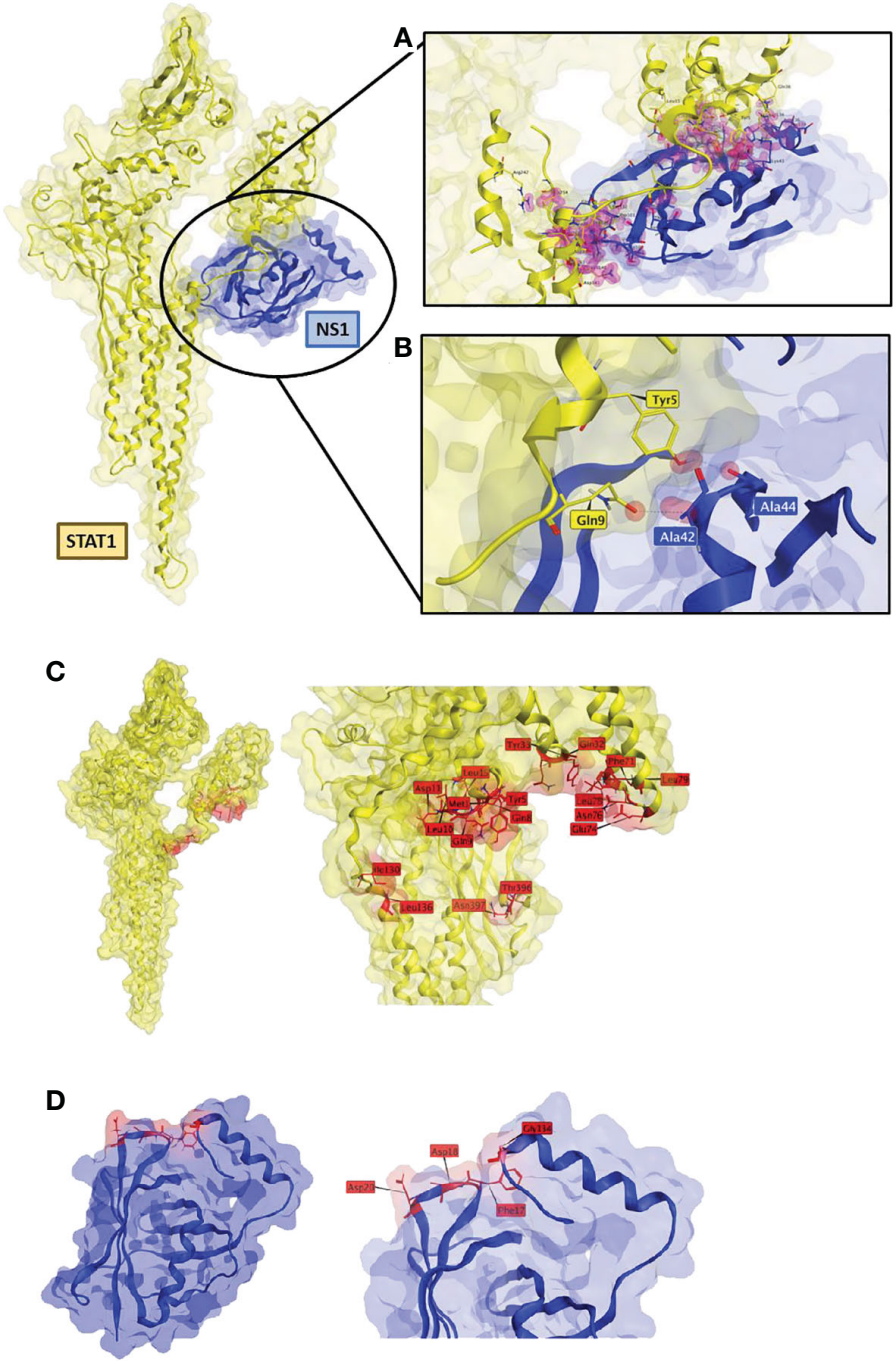
RSV-NS1 suggested binding to STAT1. Best protein-protein docked pose of STAT1 (PDB: 1YVL yellow) and RSV-NS1 (PDB: 5VJ2 blue) predicted by MOE 2022.02. **(A)** MOE Contact analysis results highlighting amino acid residues (red) involved in possible interactions between RSV-NS1 and STAT1. **(B)** Hydrogen bond interactions (dotted lines) between amino acids of RSV-NS1 (Ala42, Ala44) and STAT1 (Gln9, Tyr5). Common amino acid contacts (red) that may be responsible for the interactions between **(C)** STAT1 (PDB: 1YVL yellow) and **(D)** RSV-NS1 (PDB: 5VJ2 blue).

To further investigate the interaction seen between RSV-NS1 and STAT1, we used Molecular Operating Environment (MOE) to identify protein-protein docking between the two proteins. This produced a best scored docked pose docking score of -86.2. The triplicate runs produced the exact same pose and docking score, indicating good reproducibility of the docking algorithm. The best docked poses obtained from ClusPro and ZDOCK were examined, and it was found that RSV-NS1 interacted at a common site on STAT1, similar to the MOE predicted docked pose (**Figure 5**). To explore these interacting sites MOE contact analysis was performed.

The contact analysis on the best MOE docked pose resulted in 63 contacts between RSV-NS1 and STAT1 (**Supplementary Table S1**) (**Figure 5A**). Two hydrogen bonds were identified (**Figure 5B**) between amino acids of RSV-NS1 (Ala42, Ala44) and STAT1 (Gln9, Tyr5) representing possible protein-protein interaction (PPI) hotspots (43). A similar contact analysis was also performed on the best docked poses from ClusPro and ZDOCK. From the contact analysis output files of the three docking protocols, we identified common contact amino acid residues (**Figure 5**). The common amino acid contacts found on STAT1 (**Figure 5C**) were Tyr5, Ty33, Asn397, Asn76, Asp11, Gln32, Gln8, Gln9, Glu74, Ile130, Leu10, Leu136, Leu15, Leu78, Leu79, Met1, Phe71 and Thr396. Whereas the common amino acid contacts found on RSV-NS1 (**Figure 5D**) were Asp20, Asp18, Phe17 and Gly134.

## Discussion

Through regulation of the JAK/STAT pathway and IRG expression, type I IFNs play an important antiviral role during viral infection. To abrogate the IFN response and facilitate viral replication, viruses have developed elaborate strategies to inhibit the type I IFN JAK/STAT pathway. These strategies include (i) blocking the interaction of IFNs to receptors; (ii) downregulating or degrading components of JAK/STAT signaling pathway or interacting with its components; (iii) blocking phosphorylation of the receptor, JAKs and STATs; (iv) impairing ISGF3 formation and IFN-induced nuclear translocation and (v) inducing negative regulators, such as SOCS and cysteine-based protein tyrosine phosphatases (PTPs) (44). Understanding how viruses disrupt immune signaling gives us an advantage when developing novel therapeutics against these broad and complex pathogens. In this study, we found that while RSV-NS1 enhanced IFN-α-induced STAT1 phosphorylation, it reduced IFN-α-induced STAT1 nuclear translocation, ISRE and GAS promoter activity and downstream antiviral IRG expression as summarized in **Supplementary Figure S1**. Further analysis showed that RSV-NS1 interacted with STAT1 and reduced the association between STAT1 and the importin, KPNA1. Protein-protein docking analysis determined a plausible binding mode between RSV-NS1 and STAT1. The presence of hydrogen bonds between Gln9, Ty5 of STAT1 and Ala42, Ala44 of RSV-NS1 could be key PPIs, which should be explored in future mutagenesis studies to confirm their involvement. The amino acid contacts identified in the study on RSV-NS1 and STAT1 can serve as starting points for future studies to confirm if they are responsible for the protein-protein interactions. Taken together, this work identifies a novel mechanism by which RSV blocks anti-viral JAK/STAT signaling responses to IFN-α.

The BEAS-2B cell line was originally established as an immortalized cell line from the human bronchial epithelium (45). Therefore, it is generally recognized as a bronchial epithelial cell line and has therefore been extensively used to study respiratory diseases. However, it should be noted that BEAS2 cells have been reported to have some characteristics of mesenchymal stem cells (46).

RSV has capacity to evade the IFN response, with studies specifically indicating a prominent role for its NS1 protein in hampering antiviral immunity. We have previously shown RSV-NS1 to degrade STAT2 through proteasomal degradation (24). RSV-NS1 also reduces IFNAR1 through miR-29a expression (47). RSV-NS1 can limit IFN-α-induced IRG induction by upregulating SOCS1, which directly associates with and inhibits activity of JAKs (26). Even though RSV infection has been shown to impair type I IFN-dependent nuclear localization of STAT1 and STAT2 in mouse bone marrow derived dendritic cells, the molecular mechanism remains unknown (48). Furthermore, none of the proteins encoded by RSV have been shown to impede the nuclear translocation of STATs in human epithelial cells. Therefore, our study provides new evidence that RSV-NS1 disrupts IFN-α signaling by interacting with STAT1 and competing with KPNA1 for association with STAT1 (49). By reducing STAT1 nuclear translocation, RSV creates an optimal environment for RSV to infect and replicate in epithelial cells, thereby causing respiratory disease.

The DNA binding domain of STAT1 possesses a noncanonical nuclear localization sequence, that is recognized by KPNA1 (50). KPNA1 primarily facilitates the transportation of the ISGF3 complex to the nucleus, making it a likely target for RSV immune evasion strategies. Indeed, viruses are well known to dampen the trafficking of STAT1 by blocking its association with importins. For instance, by binding KPNA1, the VP24 protein of the Ebola virus hinders the nuclear translocation of STAT1 (51); and a recent study revealed that SARS-CoV-2 ORF6 binds to the nuclear pore complex, thus inhibiting STAT1 nuclear translocation (52). Furthermore, the 3Cpro of Foot and Mouth Disease Virus degrades KPNA1, thereby inhibiting STAT1 nuclear translocation (42). The Nsp1β of the porcine respiratory and reproductive virus prevents the nuclear translocation of ISGF3, by also triggering degradation of KPNA1 through a ubiquitin-proteasome mechanism (41). To investigate if RSV used a similar mechanism, we firstly expressed RSV-NS1 in BEAS 2b cells and the subcellular location of STAT1 was quantified by confocal microscopy. On expression of the RSV-NS1 protein there was significantly less STAT1 located in the nucleus following IFN-α treatment. This aligns with our observations that NS1 expression reduced IRG expression despite an increase in IFN-α-induced pSTAT1. While these results reveal RSV-NS1 as an inhibitor of STAT1 nuclear translocation, future studies should consider analyzing the nuclear translocation of other STATs, including STAT2. The mRNA expression of several IRGs, including *MxA*, *USP18*, *PKR* and *ISG15*, was measured here to characterize the functional antiviral output of the JAK/STAT pathway. These IRGs have several actions, MxA is well known to prevent viral replication (53); USP18 cleaves ISG15 from a range of proteins (54); PKR prevents viral protein translation (55) and ISG15 disrupts the viral budding of viruses from the cell (56, 57). This array of antiviral effects underscores the significance of viruses limiting IRG expression and effectiveness. Overall, the reduced IRG expression provides an insight into the antiviral effects of RSV-NS1 upon type I IFN signaling in epithelial cells.

In summary, we have elucidated a mechanism by which RSV-NS1 inhibits IFN-α responses by blocking STAT1 nuclear translocation. The impaired KPNA1 binding to STAT1 in the presence of RSV-NS1 reveals an evolved strategy by which RSV escapes innate antiviral immunity. By controlling the nuclear translocation of STAT1, RSV limits normal anti-viral JAK/STAT signaling, thus reducing the type I IFN response of the host cell and creating a cellular environment optimal for enhanced viral replication. These findings enhance our understanding of respiratory virus immune evasion mechanisms and reveal RSV-NS1 as a possible target for therapeutic intervention.

## Data availability statement

The raw data supporting the conclusions of this article will be made available by the authors, without undue reservation.

## Ethics statement

Ethical approval was not required for the studies on humans in accordance with the local legislation and institutional requirements because only commercially available established cell lines were used.

## Author contributions

CE: Formal analysis, Writing – original draft, Writing – review & editing, Data curation, Investigation, Methodology, Visualization. YZ: Writing – original draft, Writing – review & editing, Data curation, Formal analysis, Investigation, Methodology, Visualization. SK: Visualization, Writing – original draft, Writing – review & editing. DF: Formal analysis, Visualization, Writing – original draft, Writing – review & editing. EM: Writing – original draft, Writing – review & editing, Conceptualization, Funding acquisition, Supervision. NS: Formal analysis, Writing – original draft, Writing – review & editing, Conceptualization, Funding acquisition, Supervision.

## Supplementary material

Supplementary Figure 1 Schematic diagram of proposed mechanism. Right hand side shows normal function of the JAK/STAT pathway. On binding of IFN to the IFNR there is a change in confirmation of the cytoplasmic tails of the receptor allowing their transphosphorylation and the activation of JAKs, which leads to receptor phosphorylation. These phosphorylation sites act as docking sites for STAT1 to bind the receptor. This results in the phosphorylation of STAT1 which can bind to form a homodimer, GAF. The GAF molecule is then bound by the KPNA1 importin and transported to the nucleus through the nuclear pore complex where it binds to GAS promoter regions to induce IRG expression. The left side shows proposed action of RSV-NS1 preventing interaction of the importin with GAF, limiting translocation of GAF to the nucleus which leads to an accumulation of pSTAT1 in the nucleus and reduced IRG expression.

Supplementary Table 1 MOE contact analysis between the amino acids of RSV-NS1 and STAT1 on MOE docked output. The table is sorted from best to low contact energies. (Type: The type of contact: Hbond (DH) and Distance (D), Energy: interaction energy in kcal/mol, Distance: The distance between the centroids of the interacting atoms. When multiple interactions are aggregated into a single entry, this value is the average distance).
