# Supplementary figures and images for: Respiratory syncytial virus NS1 inhibits anti-viral Interferon-α-induced JAK/STAT signaling, by limiting the nuclear translocation of STAT1
Source: Front Immunol. 2024 Jun 13;15:1395809. doi: 10.3389/fimmu.2024.1395809 (PMC11208467; doi:10.3389/fimmu.2024.1395809)

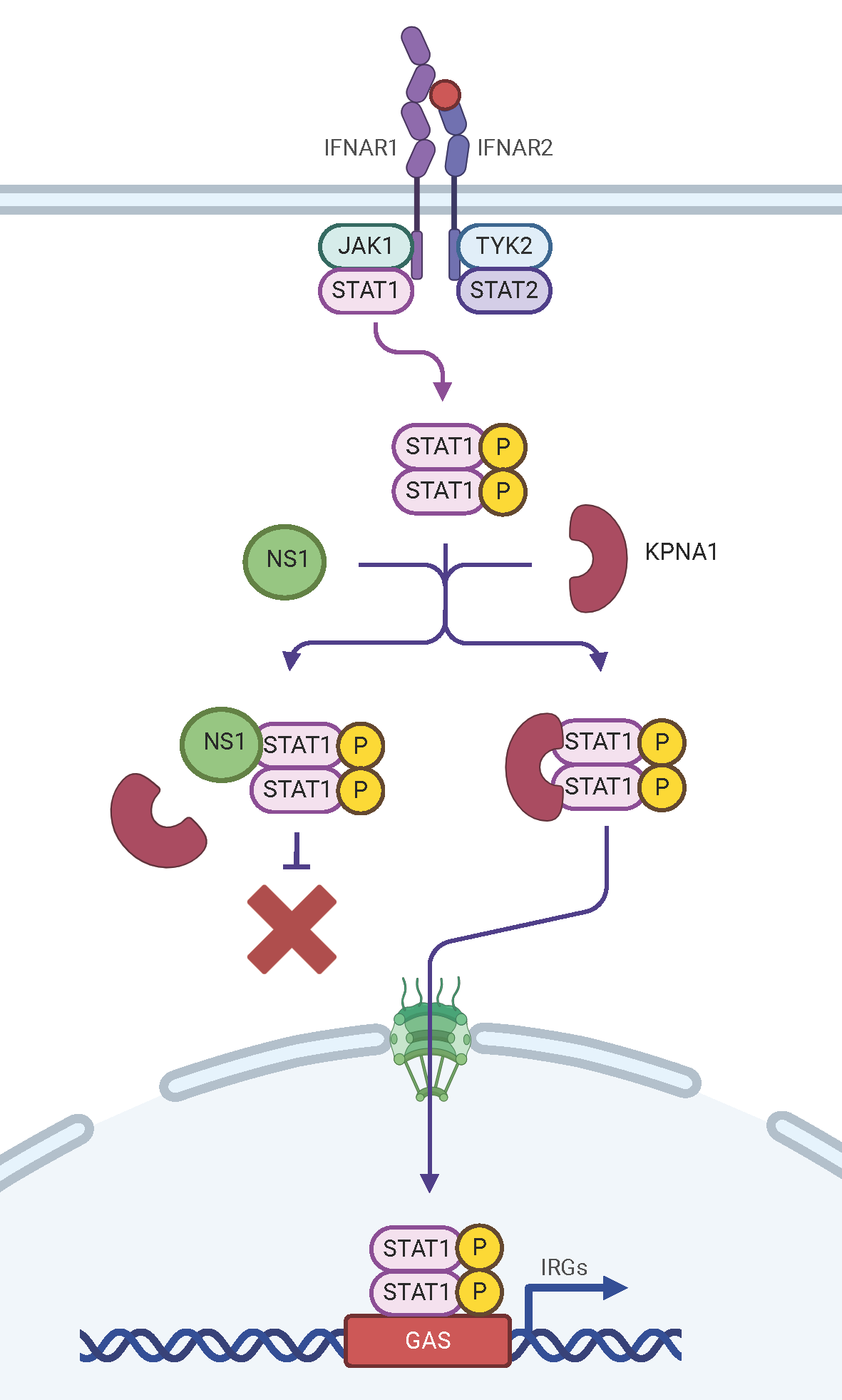

Supplement: Supplementary Figure 1 — Schematic diagram of proposed mechanism. Right hand side shows normal function of the JAK/STAT pathway. On binding of IFN to the IFNR there is a change in confirmation of the cytoplasmic tails of the receptor allowing their transphosphorylation and the activation of JAKs, which leads to receptor phosphorylation. These phosphorylation sites act as docking sites for STAT1 to bind the receptor. This results in the phosphorylation of STAT1 which can bind to form a homodimer, GAF. The GAF molecule is then bound by the KPNA1 importin and transported to the nucleus through the nuclear pore complex where it binds to GAS promoter regions to induce IRG expression. The left side shows proposed action of RSV-NS1 preventing interaction of the importin with GAF, limiting translocation of GAF to the nucleus which leads to an accumulation of pSTAT1 in the nucleus and reduced IRG expression. [file Image_1.tif]
