# Supplementary material for: Respiratory syncytial virus NS1 inhibits anti-viral Interferon-α-induced JAK/STAT signaling, by limiting the nuclear translocation of STAT1
Source: Front Immunol. 2024 Jun 13;15:1395809. doi: 10.3389/fimmu.2024.1395809 (PMC11208467; doi:10.3389/fimmu.2024.1395809)
Supplement: Supplementary Table 1 — MOE contact analysis between the amino acids of RSV-NS1 and STAT1 on MOE docked output. The table is sorted from best to low contact energies. (Type: The type of contact: Hbond (DH) and Distance (D), Energy: interaction energy in kcal/mol, Distance: The distance between the centroids of the interacting atoms. When multiple interactions are aggregated into a single entry, this value is the average distance). [file Table_1.docx]

| **Type** | **1YVL (STAT1) AA** | **5VJ2 (NS1) AA** | **Energy** | **Distance** |
| --- | --- | --- | --- | --- |
| D | Leu79 | Asn19 | -2.48 | 3.83 |
| D | Asn397 | Leu104 | -1.93 | 3.7 |
| D | Met1 | Pro139 | -1.88 | 3.84 |
| D | Gln8 | Met93 | -1.72 | 4 |
| D | Leu136 | Pro101 | -1.7 | 3.25 |
| D | Asn261 | Leu104 | -1.57 | 4.16 |
| D | Met1 | Lys43 | -1.39 | 4.07 |
| DH | Tyr5 | Ala44 | -1.32 | 4.01 |
| D | Met135 | Gln100 | -1.07 | 3.91 |
| D | Tyr5 | Phe135 | -0.81 | 3.95 |
| D | Ile130 | Thr31 | -0.78 | 4.04 |
| DH | Gln9 | Ala42 | -0.75 | 4.17 |
| D | Gln258 | Gln100 | -0.67 | 3.95 |
| D | Leu15 | Glu94 | -0.48 | 4.29 |
| D | Gln32 | Met93 | -0.38 | 4.18 |
| D | Phe71 | Asn19 | -0.31 | 4.38 |
| D | Ala254 | Gln100 | -0.3 | 3.89 |
| D | Leu136 | Asn102 | -0.29 | 4.07 |
| D | Gln36 | Gly134 | -0.28 | 3.83 |
| D | Leu136 | Leu6 | -0.28 | 3.79 |
| D | Gln258 | Asn102 | -0.24 | 3.85 |
| D | Asn76 | Asp18 | -0.23 | 3.94 |
| D | Leu78 | Asp20 | -0.22 | 3.95 |
| D | Gln8 | Glu94 | -0.19 | 3.9 |
| D | Thr396 | Asn76 | -0.17 | 4.44 |
| D | Gln8 | Leu95 | -0.14 | 3.84 |
| D | Met1 | Ala44 | -0.12 | 4.44 |
| D | Asp143 | Asn102 | -0.11 | 4.13 |
| D | Leu15 | Leu95 | -0.08 | 4.33 |
| D | Thr396 | Thr78 | -0.07 | 4.3 |
| D | Tyr5 | Met92 | -0.04 | 4.03 |
| D | Gln32 | Glu91 | -0.04 | 4.38 |
| D | Tyr5 | Leu41 | 0 | 4.15 |
| D | Gln8 | Glu91 | 0 | 4.5 |
| D | Gln9 | Leu41 | 0 | 3.79 |
| D | Glu74 | Asp18 | 0 | 4.2 |
| D | Asp137 | Ser5 | 0 | 4.45 |
| D | Arg242 | Gln100 | 0 | 4.22 |
| D | Thr396 | Asp106 | 0 | 4.4 |
| D | Gln8 | Met92 | 0.01 | 4.07 |
| D | Glu74 | Asn19 | 0.01 | 4.25 |
| D | Ser132 | Thr31 | 0.03 | 4.22 |
| D | Gln36 | Asp136 | 0.07 | 4.37 |
| D | Glu74 | Leu16 | 0.11 | 3.76 |
| D | Leu10 | Leu95 | 0.14 | 4.01 |
| D | Asp11 | His97 | 0.23 | 3.73 |
| D | Gln32 | Gln14 | 0.23 | 4.23 |
| D | Leu136 | Ser5 | 0.36 | 4.43 |
| D | Gln139 | Gln100 | 0.39 | 4.06 |
| D | Gln9 | Met92 | 0.41 | 3.9 |
| D | Gln36 | Phe135 | 0.46 | 4.28 |
| D | Gln9 | Leu95 | 0.63 | 4.23 |
| D | Tyr33 | Asn19 | 0.64 | 4.21 |
| D | Asp257 | Leu104 | 0.66 | 4.06 |
| D | Asn76 | Asp20 | 0.77 | 3.81 |
| D | Gln258 | Leu104 | 0.78 | 4.3 |
| D | Gln139 | Asn102 | 0.85 | 4.09 |
| D | Glu74 | Phe17 | 0.9 | 3.67 |
| D | Lys140 | Ser5 | 1.1 | 3.99 |
| D | Gln258 | Gly103 | 1.16 | 3.91 |
| D | Met1 | Ala42 | 1.53 | 3.86 |
| D | Lys140 | Asn102 | 1.54 | 3.82 |
| D | Asn76 | Asn19 | 1.82 | 3.82 |
